# Atlantoaxial Instability in the Course of Rheumatoid Arthritis in Relation to Selected Parameters of Sagittal Balance

**DOI:** 10.3390/jcm13154441

**Published:** 2024-07-29

**Authors:** Robert Wróblewski, Małgorzata Mańczak, Robert Gasik

**Affiliations:** 1 Department of Neuroorthopedics and Neurology Clinic and Polyclinic, National Institute of Geriatrics, Rheumatology and Rehabilitation in Warsaw, 1 Spartanska Street, 02-637 Warsaw, Poland; 2 Department of Gerontology, Public Health and Didactics, National Institute of Geriatrics, Rheumatology and Rehabilitation in Warsaw, 1 Spartanska Street, 02-637 Warsaw, Poland

**Keywords:** rheumatoid arthritis, sagittal balance, atlantoaxial instability

## Abstract

**Background:** Atlantoaxial instability is the most common cervical instability in patients with rheumatoid arthritis (RA). Its course may differ in different patients and may have different degrees of severity and symptoms. **Methods:** There are a number of studies on systemic factors associated with the development of this instability, but there are few publications in the scientific literature on the influence of biomechanical factors on the development of cervical instability. One of the areas that allows the study of biomechanical factors influencing spine pathologies is the analysis of sagittal balance using radiological parameters. The study of radiological parameters of sagittal balance has contributed to understanding the pathology of selected spine diseases and is currently an indispensable tool in planning surgical treatment. **Results:** The presented study, conducted on a group of RA patients with cervical instability, was performed to look for a relationship between C1–C2 instability and sagittal balance parameters. **Conclusions:** Among the examined selected parameters, a statistically relationship between C1–C2 instability and the Cobb angle C1–C7 and OD-HA parameters has been found. This confirms the need for further in-depth research on this areas.

## 1. Introduction

In the course of rheumatoid arthritis (RA), the third-most frequently affected area of the musculoskeletal system after the joints of the hands and feet, are the joints of the cervical spine [1,2,3]. According to A. Garrod, who in 1890 in *A Treatise on Rheumatism and Rheumatoid Arthritis* was the first to describe the impact of rheumatoid arthritis on the cervical joints, it occurs in over 35% of patients, and according to more recent data, radiological changes are observed in over 80% of patients [3,4,5,6,7]. The joints of the cervical spine are rich in synovial membrane and are therefore susceptible to the autoimmune inflammatory process characteristic of rheumatoid arthritis. The inflammatory process of the synovium of a progressive and recurrent nature leads to its destruction and dysfunction [8]. Damaged joints of the cervical spine lose their congruence, leading to non-physiological displacements of the vertebrae and the surrounding tissues as well as to pressure on the spinal cord, blood vessels and nerve roots. Instability may be asymptomatic, it may be characterized by pain, most often in the occiput area, and progressive pressure may lead to myelopathy, radiculopathy, and in extreme cases to disturbances in the functions of vital centers and death [1,2].

The most common type of instability in the course of RA in the cervical spine is atlantoaxial subluxation (AAS) [1,4]. In scientific articles, the diagnosis of AAS among all cases of instability ranges from 10% to 88%, that of SAS (subaxial subluxation) 20%, and that of CrS (cranial settling) instability of 11% [4,5,6,7,9,10,11,12,13,14].

The atlantoaxial joint is the most mobile joint in the human body, characterized by a lack of natural bone stabilization and responsible for 50% of the entire rotational movement of the neck, for 5% of the lateral tilt, and from 10% to 20% of flexion and extension [10,15].

**Figure 1:**
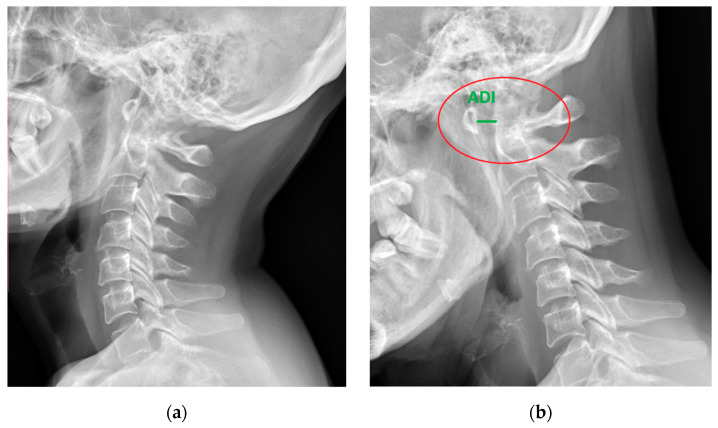
Lateral X-ray of the neck of a 49-year-old patient with rheumatoid arthritis and atlantoaxial subluxation (red circle), (**a**) neutral position, and (**b**) flexion. ADI—atlantodental interval (green).

**Figure 2:**
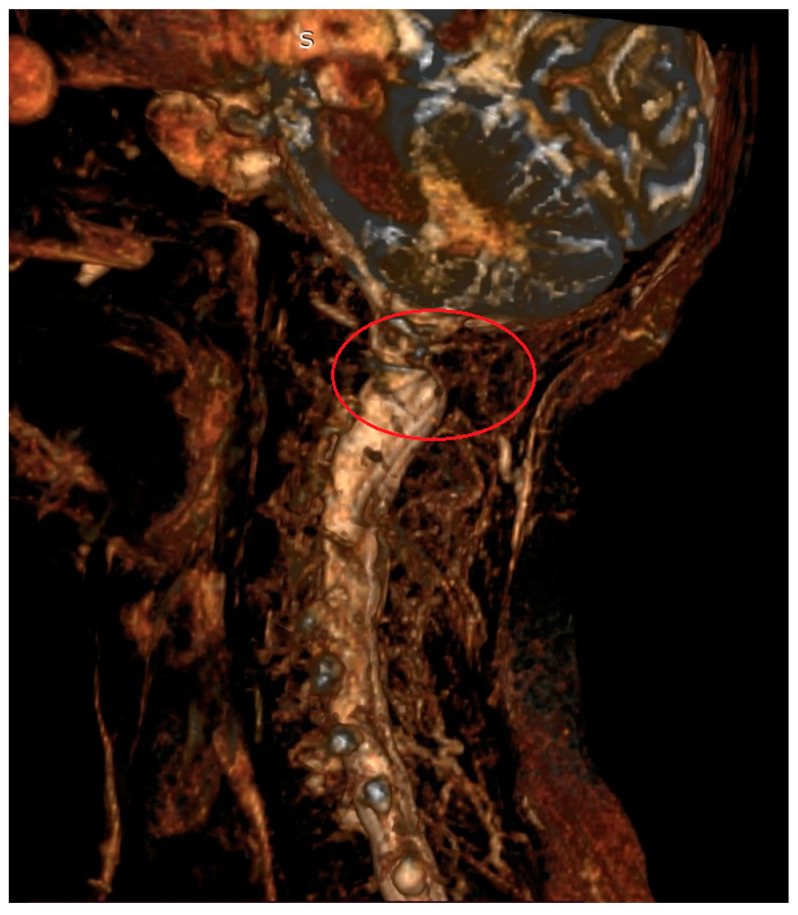
Magnetic resonance imaging (3D) of the cervical spine in a 67-year-old patient with AAS due to rheumatoid arthritis. Visible compression of neural structures in the area of the C1–C2 segment (red circle).

The C1–C2 segment is composed of two vertebrae, atlas and axis, with a significantly different structure from the other cervical vertebrae, and a ligament apparatus, which includes the cruciate ligament complex and allows unprecedented mobility in relation to other spinal joints [10,16]. The location of the C1–C2 segment at the level of the transition of the medulla oblongata into the spinal cord and the characteristic course of the vertebral arteries in the upper cervical spine contribute to the complexity and seriousness of the symptoms [3,17,18,19] (Figure 1 and Figure 2).

Instability of the cervical spine in rheumatoid arthritis is a serious clinical problem. The inflammatory process in RA attacks the joints of the cervical spine, leading to their deformity. Cervical deformities can manifest themselves in many forms that are relevant in case of surgical treatment. These may include the aforementioned joint instability and associated foramen and canal stenosis as well as adjacent segment disease (ASD) associated with post-inflammatory ankylosis or previous surgical treatment, fractures, osteoporosis, and regional deformities [2,3,4,7,8]. The decision for surgical treatment should be made before neurological or vascular damage occurs [3,7].

**Figure 3:**
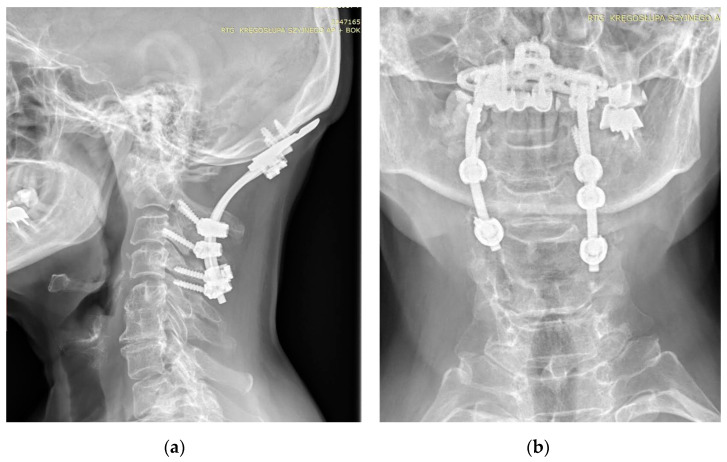
AP (**a**) and lateral (**b**) X-rays of the neck of a 67-year-old patient with rheumatoid arthritis and atlantoaxial subluxation, CrS, and ankylosis C5-C6. X-ray showing occipitocervical fusion with C1 laminectomy. Due to anatomical deformities in the course of RA, asymmetric screw placement was possible.

The first procedure considered in the setting of SAA is a C1–C2 fusion. The method evolved from the techniques involving wiring and grafting introduced by Gallie (1939) and their modifications. The most commonly employed methods at this time involve C1/2 transarticular screw fixation or a combination of C1 lateral mass screw and C2 pars or pedicle screw fixation [20,21,22]. Various types of these techniques were described by Magerl (1986), Goel and Laheri (1994), and Harms and Melcher (2001), and then were modified by various authors [23,24,25,26]. In some cases, occipitocervical fusion may be used [27,28]. This technique may be preferred in cases where AAS has progressed to cranial settling, canal stenosis, and increased risk of ASD [29]. Grob (1991) was the first to describe the technique currently used, which includes an occipital plate and cervical screw implantation [30]. The cervical stabilization techniques used depend on whether the lesions at C1–C2 are repositionable or not, the extent and type of lesions in adjacent segments, anatomical considerations, and operator experience (Figure 3).

Due to the potentially lethal nature of the disease and the fact that the exact etiology of rheumatoid arthritis is unknown, great importance is attached to understanding the factors predisposing to its development and consequences. The course of the disease and instability vary from patient to patient. In some people, instability and the consequences associated with it develop more slowly, in others more quickly. A number of multidisciplinary studies have been carried out focusing on understanding the systemic factors predisposing to its development [31,32,33,34,35,36]. An issue that has in recent years significantly contributed to expanding knowledge of spine pathology and led to progress in surgical treatment is the issue of mapping the individual characteristics of patients’ spine biomechanics using radiological parameters of sagittal balance [37,38,39]. However, in the area of cervical spine instability in patients with rheumatoid arthritis, there are still many issues that require clarification.

## 2. Materials and Methods

This study was retrospective in nature. The study group consisted of patients hospitalized at NIGRiR clinics with diagnosed RA according to the criteria developed by the ACR and EULAR [40]. The study was approved by the hospital bioethics committee and all patients consented to it.

Due to the history of the underlying disease, the patients underwent functional X-ray examinations of the cervical spine [41,42,43]. The initial inclusion criterion was the diagnosis of cervical instability (AAS, SAS, or CrS) in these patients. AAS instability has been defined as the ADI interval, i.e., the distance between the posterior edge of the anterior arch and the anterior edge of the axial dense, equal to or greater than 3.0 mm [11]. The definition of SAS (subaxial subluxation) in C2 has been defined as the displacement of the upper edge of the posterior wall of the lower vertebra in relation to the lower edge of the posterior wall of the upper vertebra equal to or greater than 2 mm [32,44]. A third type of instability CrS (cranial settling) is manifested migration of the odontoid into the foramen magnum [45,46]. Patients diagnosed with instability were referred for neuro-orthopedic consultation. Patients meeting the criteria for instability of the cervical region had extended diagnostics with a postural examination of the spine [47]. This examination is dedicated to patients for whom a spinal surgery is considered or planned. Exclusion criteria included a clear history of other cervical spondylosis or cervical spine injury and previous spine surgery and also included patents with a severe neck infection, tumor, congenital deformity and other diseases that could lead to instability of the cervical spine, and patients with a serious disability that prevented radiological examination in a standing position without support.

Research focused on finding a relationship between instability at the C1–C2 level and selected parameters of sagittal balance due to the highest incidence of instability at this level [1,4,12,48].

The results of AAS patients have been assessed radiologically. In the assessment of the functional examination of the cervical spine, the ADI (atlantodental interval) distance expressed in millimeters has been taken into account. During postural examination, parameters of the sagittal balance of the cranial, cervical, thoracic, lumbar and pelvic sections have been determined in each of the subjects. The parameters selected for the study allow for the analysis of multidirectional reference points of the biomechanical chain of the human skeletal system, reflecting the essence of the topic of the work. They include: OI, OS (in the W. Zhu mod.), COG-C7SVA, C2–C7SVA, C7SVA HD, OD-HA (mm), OD-HA (st), McGS, Cobb angle C1–C7, Cobb angle C0–C2, Cobb angle C1–C2, Cobb angle C2–C7, C7S (in some patients T1S), ThK, LL, PI, SS and PT [9,49,50]. The radiological parameter definitions are presented in Table 1.

Radiological examinations were performed on the Carestream DRX Evolution—Health protocol. They were performed under constant conditions (by one radiologist) after adopting positions dedicated to postural examinations [47]. The obtained results were then subjected to statistical analysis. Statistical analyses were carried out using STATISTICA v.13.1 (Statsoft; Dell Inc., Tulsa, OK, USA, 2016). The compliance of the distributions of the studied quantitative variables with normal distribution was examined using Kolmogorov–Smirnov tests. The distributions deviated from normal. Continuous variables are presented as median (Me) and interquartile range (IQR). Spearman’s correlation coefficients were used to assess the existence of relationships between quantitative variables. The limit of statistical significance was *p* < 0.05.

## 3. Results

In sum, 47 patients were included in the study: 40 (85%) women and 7 (15%) men. The median age in the analyzed group was 66 (IQR: 58–74) years. The median duration of the disease was 20 (12–30) years. In the study, AAS had been diagnosed in 27 patients. The mean age of these patients with AAS was 64 years, and those without instability 70.5 years.

Initially, 54 patients qualified for the study, of which 47 remained. One person was excluded during the study due to changes that made it impossible to perform a postural X-ray examination in accordance with the set criteria. The rest of those excluded did not complete the diagnostic process due to the COVID-19 pandemic, namely, by interruptions in the operation of the clinic, considerations for patient safety and restrictions of patient access to medical services at that time. The distribution of identified instabilities is presented in Table 2.

Out of the 47 patients, 20 patients had instability at one level, 19 patients at two levels, 4 patients at three levels, 3 patients at four levels, and 1 patient at five levels. In the studied group of patients, the largest percentage were patients with instability at the C1–C2 level—31%.

Table 3 shows Spearman’s correlation coefficients between ADI and sagittal balance parameters in the entire study group and in the subgroup of patients with instability at the C1–C2 level. A statistically significant, weak negative correlation was found between the C1–C7 (in degrees) value and ADI in the entire study group (rho = −0.293; *p* = 0.046). Weak positive correlations (bordering on statistical significance) were also found between ADI and absolute values of OD-HA (abs OD-HA), expressed in millimeters and degrees. The Spearman coefficient values were: rho = 0.275; *p* = 0.065 for abs OD-HA (mm) and rho = 0.287; *p* = 0.053 for abs OD-HA (in degrees).

A weak negative correlation was also found between ADI and Cobb angle C2–C7 (in degrees): rho = −0.256; *p* = 0.082. The results are presented in Table 3.

Correlations between OD-HA and C7 SVA HD angle values were also analyzed. A statistically significant and strong positive correlation was found between the values of OD-HA angles expressed in both millimeters and degrees and the values of C7 SVA HD (mm) (Table 4).

**Figure 4:**
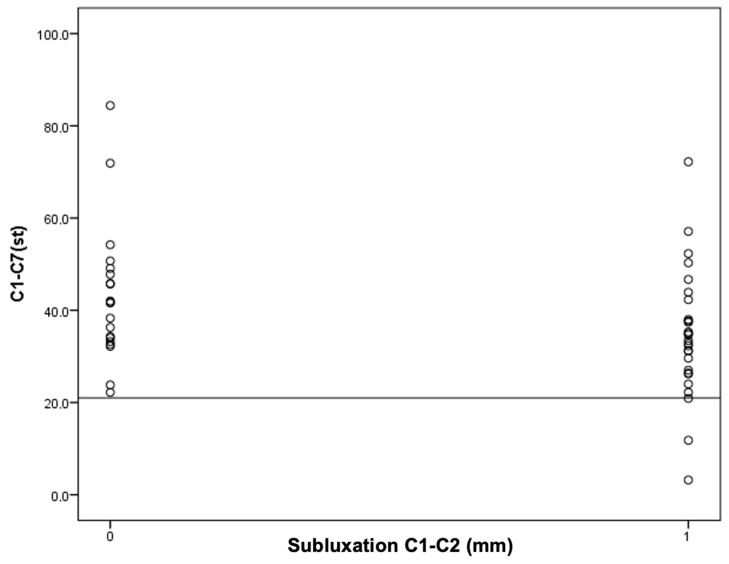
An attempt to determine the cutoff value for C1–C2 instability in relation to the C1–C7 Cobb angle.

**Figure 5:**
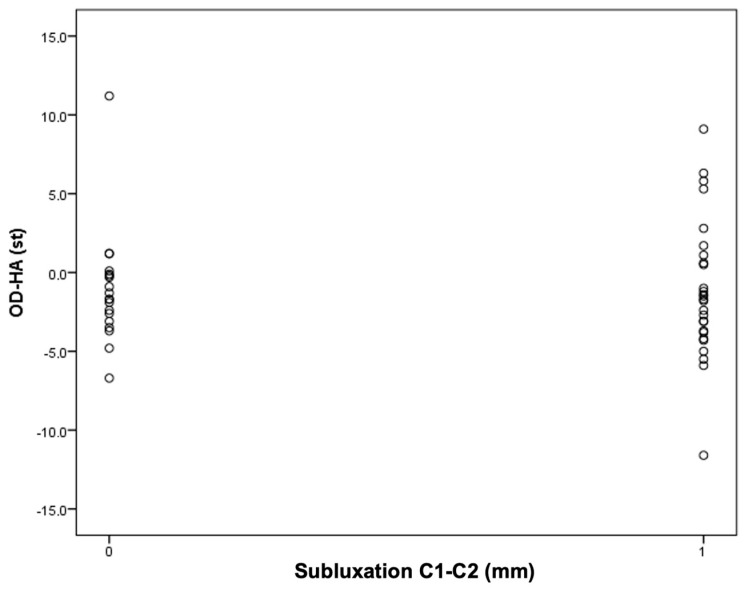
An attempt to determine the cutoff value for C1–C2 instability depending on the OD-HA value.

Next, an attempt was made to find the cutoff points for the occurrence of C1–C2 instability for the OD-HA value and the C1–C7 Cobb angle (Figure 4 and Figure 5).

It was found that in the group of patients with C1–C2 subluxation, three patients (11%) had a Cobb angle less than 21 degrees. However, in the group of patients without C1–C2 subluxation, the Cobb angle values for all patients were higher than 21 degrees. In other words, all patients with a C1–C7 Cobb angle smaller than 21° had C1–C2 subluxation.

It was found that among patients with an OD-HA value above +2 degrees, the majority have C1–C2 subluxation: five people (19%) in the group with instability versus one person (5%) in the group without instability.

## 4. Discussion

Despite the significant progress in work on the sagittal balance of the human cervical spine observed over the last 10 years, which has allowed for not only the determination of sagittal alignment parameters but has also led to proposals for a range of normative values and a description in scientific publications of the relationship between the sagittal alignment of the neck and both disability and myelopathy, the strategy to achieve “correct neck positioning” is still subject to discussion [51,52,53].

The unification of terminology has made it possible to create classifications of cervical deformities that take into account the character and the location of the peak of the deformity in relation to the value of selected sagittal balance parameters [29,54,55]. The resulting classifications of both Ames et al. and Koller, using static X-ray examinations, as well as those of Kimm et al., using dynamic examinations, have provided a basis for standardizing the strategy of surgical planning [56,57,58,59]. These classifications use sagittal balance parameters such as C2–C7SVA, CBVA, T1S, CL, and SRS-Schwab Classification (PI, PT, C7SVA), which have been confirmed to affect the quality of life of patients with cervical diseases [9,51,60,61,62,63].

In the scientific literature, several works regarding sagittal balance in RA, such as those of Masamoto K et al., Mochizuki T et al., Rodrigo K et al. have been published [64,65,66]. However, publications on the role of sagittal balance in the instability of the cervical spine in RA patients appear only when analyzing the results of surgical treatment of patients. On this basis, it can be noted, among other things, that the relationship between the increase in the C1–C7 lordosis angle and the reduction in instability that has been described in the statistical analysis in the discussed work is complex. This is evidenced by research, including that of Huang et al. and Ishii et al., or the work of Yoshimota et al., which have all shown how excessive surgical correction of the C1–C2 angle in operated patients with RA may cause the loss of cervical lordosis and consequently the development of postoperative SAS [67,68,69]. In turn, in the work of Wu et al., it has been suggested that insufficient correction of the C0–C2 angle may also lead to the development of adjacent segment syndrome and SAS [70]. A certain morphological and functional distinctiveness in RA patients has also been noticed. Comparing surgical C1–C2 anastomoses in patients with and without RA, Ito et al. noted a strong correlation between Cobb’s angle C1–C2 and Cobb’s angle C2–C7 in RA patients. In the study group, it was found that postoperative C1–C2 reductions in the C2–C7 angle, followed by anterior subluxation and eventual neurological deterioration, occurred only in RA patients [71].

When planning surgical treatment, it should also be noted that in the case of RA patients, characterized by poor bone quality, selected complications such as destabilization, implant subsidence, adjacent segment syndrome, and lack of fusion have a higher risk of potential irreversibility [9,72,73,74].

In this study, which has searched for a relationship between the ADI value and the cranial, thoracic, lumbar and pelvic parameters, the relationship between the Cobb angle C1–C7 (negative relationship) and abs OD-HA (positive relationship) with the ADI value has been shown. These results may suggest a relationship between these parameters and the development of AAS. It is worth noting that these parameters are of a different nature.

The first is a regional parameter covering one anatomical and functional section of the spine. The C1–C7 Cobb angle is related to the distribution of force vectors acting on the vertebral bodies depending on the inclination angle [38]. Based on the method of measuring cervical lordosis angles proposed by Harrison et al., it can be assumed that each of the component angles may constitute a separate value, important for the development of instability [75]. The topic of the work prompted directing more attention to the distinctive anatomical structure of the basic functional unit of the C1–C2 segment and its biomechanical properties. Among the differences that contribute to explaining the obtained results of statistical analysis are the mechanisms described by Kapandji et al. [15]. These include the fact that in the C1–C2 joint, movement takes place on the convex oval articular surfaces of the paired atlantoaxial joints. When the ligamentous apparatus is preserved, the elasticity of the ligaments protects the joints of the C1 and C2 vertebral bodies against permanent displacement. In cases of damage to the ligaments of the cruciate complex, the highest point of contact of the articular surfaces in both joints may be exceeded simultaneously, with shear forces predominating and both symmetrical lower C1 joints sliding forward and downwards, which manifests itself in an increase in the ADI distance and the retraction of the axial dense into the spinal canal. This movement may intensify especially when the C1–C2 angle decreases, together with the C1–C7 angle, because then the stabilizing role of the posterior ligaments, including the nuchal ligaments and the stretched occipital muscles and joint capsules, is smaller. In addition, as the neck tilts forward, the head’s center of gravity (COG) shifts forward, which leads to changes in the distribution of force vectors acting on the cervical vertebrae, similar to the changes described in Rossouli’s work [76].

This shift may not be important in the neutral position. However, if the patient, by tilting the head forward, shifts the COG relative to C2SVA, and happens to suffer damage to the ligaments of the cruciate complex—as a result of the changed distribution of forces—this causes the oval lower articular surfaces of C1 to slide from the upper articular surfaces of C2, which in turn leads to the displacement of the axial dense towards the spinal canal [15]. The obtained results of statistical tests suggest that increasing the Cobb angle C1–C7 protects against both lower C1 joints simultaneously crossing this critical point of the upper C2 joints.

The second parameter obtained in the analysis, one of a global character, is the reference to the deviation in the dens axis relative to the sagittal axis of the SVA passing through the heads of the femurs [9,77,78]. The abs parameter of OD-HA captures J. Duboussete’s idea of an “economic cone”. According to J. Duboussete, sagittal equilibrium is a balanced ratio of all curvatures of the spine (kyphosis, lordosis) that is meant to maintain the position of the body in space against the forces of gravity economically using as little energy as possible [79]. According to J. Duboussete’s theory, the sagittal alignment of spinal curvature must be contained within an “economic cone” and a deviation in any direction from the SVA drawn from the feet to the cranium requires an increased expenditure of energy when returning to the initial position. The return to the initial position even with significant deviation is possible in a healthy ligamentous and muscular apparatus. However, in patients with RA, movement within the FSU (functional spinal unit) in the “elastic” zone that limits the passive osteochondral stabilization mechanism is irreversibly impaired [39,80,81]. Hence, it can be assumed that in patients with RA, global balance parameters, reflecting changes in the FSU above the C7 vertebra, may play a very important role, as well as regional ones. The reference points of the lower part of the axial skeleton, including the pelvis, are the center of the heads of the femurs and the posterior edge of the upper plate of the sacrum. Physiologically, the COG of the head lies above the center of the C2 body and the femoral heads. Therefore, the course of the COG SVA, C2SVA, and C7SVA HD axes and the sagittal vertical axis passing through the heads of the femurs (hip sagittal vertical axis—HSVA) reflect global balance. The scope of translation between the abovementioned axes and their position in relation to the pelvis can be used to determine global balance disorders. In practice, it has been confirmed that the translation of the SVA may have clinical consequences, e.g., the displacement of the C2SVA relative to the C7SVA HD is associated with a decrease in the Health-Related Quality of Life (HRQoL) index of patients [82,83,84].

It is worth noting that the obtained statistical analysis has not shown a direct relationship between the basic global sagittal balance parameter C7SVA HD and the development of AAS instability. The results of the analysis showed, however, in the group of RA patients an interesting, statistically significant relationship between the values of the OD-HA parameter and the C7SVA HD parameter, i.e., a parameter that takes into account the direction of the deviation (Table 4).

This may confirm the importance of the C7 SVA HD parameter in sagittal balance. The C7/SFD parameter (Barrey’s index) is a parameter that reflects the relationship between the SVA running through the heads of the femurs and the posterior edge of the S1 plate in relation to the C7SVA HD [85]. Therefore, it is a parameter reflecting the relationship between the position of the C7 vertebral body in relation to the pelvis. The OD-HA parameter allows these relationships to be transferred to the level covering the cervical section, and the statistically strongly positive result between C7SVA HD and OD-HA may not be accidental. However, conclusions should be drawn with caution and this relationship and the previous results should be subject of further research on sagittal balance in patients with RA.

The results of this work may indicate that the search for one “golden parameter” defining the strategy of “correct neck positioning” may not be sufficient. The obtained results suggest that in the development of C1–C2 instability, in addition to ligament damage caused by the inflammatory process, an additional biomechanical condition should be met that facilitates the lower C1 joint crossing the highest critical point of the convex surface of the upper C2 joint. Such a situation may be influenced, among other factors, by the C1–C7 Cobb angle: the degree of inclination of the C2 vertebra in relation to the horizontal plane, the angle between the C1 and C2 vertebrae, the inclination of the C1 vertebra or the destruction of the convex shape of the upper C2 joint. However, in addition to local changes, translational displacement of C2SVA in relation to the SVA passing through the femoral heads and relative to the pelvis may also contribute to the development of AAS.

The obtained results encourage continued work. The results of the current study, in which it has been observed that in the study group, all patients with AAS have a Cobb angle less than 21 degrees and that most of the patients with AAS have an OD-HA angle equal to or greater than +2 degrees may not have statistical significance, but do deserve attention (Figure 3 and Figure 4).

It is noteworthy that the value of +2 coincides with the range of the norm described in the work of Ambiele et al. regarding this parameter, i.e., from +2 to −5, which may confirm its clinical significance in treatment planning [82].

It is necessary to enlarge the group of subjects in order to find the cutoff values of the tested parameters for the development of instability. The obtained observations may contribute to the identification of a group with a potential for the development of severe AAS and translate into a reduction in the risk of postoperative complications of the development of AAS.

Limitations of this study include the sample size: it requires extension to a larger group of examined patients. The study did not include patients whose disease was advanced enough to prevent them from assuming a free upright position without support. The X-ray examinations were performed on film in a 2D projection, while the OD-HA parameter is assumed to be a 3D parameter, so although it is described in publications also for the assessment of 2D X-rays, inaccuracies cannot be ruled out.

## 5. Conclusions

Statistical analysis has shown a weak negative correlation between ADI (C1–C2 instability) and Cobb angle C1–C7. Weak positive correlations (bordering on statistical significance) have also been found between ADI (C1–C2 instability) and absolute values of OD-HA (abs OD-HA). A strong positive correlation has also been demonstrated between global OD-HA and C7SVA parameters.

## Figures and Tables

**Table 1:** Definitions of selected parameters of sagittal balance of the cranial, cervical, thoracic, lumbar and pelvic sections [9,49,50].

**Skull**	
McGS	the angle between the line connecting the back edge of the pallatum with the caudal edge of foremen magnum and the horizontal line
OI	the angle between the line connecting the center of the skull and the center of foramen magnum and the line perpendicular to the foramen magnum
OT	the angle between the line directed through the center of the skull and the center of the foramen magnum and SVA
OS	the angle between the line parallel to the foremen magnum and the horizontal line (according to the W. Zhu modification)
**Cervical**	
COG-C7 SVA	the distance between the SVA—directed from COG—and the center of the C7 (vertebra)
C2–C7 SVA	the distance between the SVA—directed from the C2 vertebra—and the SVA directed from the center of C7
Cobb angle C0–C2	the angle between the McRea line and the lower surface of the C2 vertebra
Cobb angle C1–C7	the angle between C1 and the lower surface of the C7
Cobb angle C2–C7	the angle between the lower surface of C2 and C7
C7S	the angle between the upper surface of the C7 vertebra and the horizontal line
**Thoracic, lumbar, sacral section and the pelvis**	
ThK	the angle between the upper Th1 plate and the lower Th12 plate
T1S	the angle between the upper plate of Th1 and the horizontal line
LL	the angle between the upper L1 plate and the upper S1 plate
SS	the angle between the upper S1 plate and the horizontal line
PT	the angle between the upper plumb line from the femur head center and the center point of the superior sacrum endplate surface
**Global**	
C7SVA HD	the horizontal distance between the C7 SVA and the back edge of upper S1 plate
ODHA (degree)	the angle between the SVA directed through the center of the femur heads
ODHA (mm)	line connecting the center of the femur heads with the axis dense

**Table 2:** Number of cases and percentage of recognized instability.

Type of Instability	Cases of Instability	Percentage
CrS	3	3.4%
C1–C2	27	31%
C2–C3	10	11.5%
C3-C4	15	17.2%
C4-C5	21	24%
C5-C6	9	10.3%
C6-C7	2	2.3%

**Table 3:** The relationship of AAS with selected basic parameters of sagittal balance (cranial, cervical, thoracic, lumbar and pelvic). Coefficient values significant at *p* < 0.05 are marked in red and coefficient values close to statistical significance at *p* < 0.05 are marked in blue.

Assessment of the Relationship between Variables, Spearman Correlations				
			ADI (mm)	ADI (mm)—Only among Patients with Subluxation
Spearman rho	COG-C7SVA (mm)	rho	−0.082	0.087
		*p*	0.585	0.667
	C2–C7 SVA (mm)	rho	−0.203	−0.203
		*p*	0.172	0.310
	C7 SVA (mm)	rho	0.105	−0.011
		*p*	0.489	0.956
	OD-HA (mm)	rho	−0.107	0.097
		*p*	0.452	0.630
	abs OD-HA (mm)	rho	0.275	0.097
		*p*	0.065	0.528
	OD-HA (degree)	rho	−0.123	0.199
		*p*	0.423	0.329
	abs OD-HA (degree)	rho	0.287	0.073
		*p*	0.053	0.653
	OI (degree)	rho	−0.077	0.005
		*p*	0.618	0.982
	OS (degree) (w mod. W. Zhu)	rho	−0.097	0.034
		*p*	0.530	0.876
	OT (degree)	rho	0.016	0.015
		*p*	0.917	0.946
	McGS (degree)	rho	0.006	0.243
		*p*	0.969	0.223
	C1–C7 (degree)	rho	−0.293	−0.050
		*p*	0.046	0.805
	C0-C2 (degree)	rho	0.108	0.095
		*p*	0.470	0.636
	C2–C7 (degree)	rho	−0.256	0.102
		*p*	0.082	0.612
	C7S (degree)	rho	−0.167	−0.014
		*p*	0.261	0.944
	T1S (degree)	rho	−0.122	−0.026
		*p*	0.493	0.911
	ThK (degree)	rho	−0.198	−0.042
		*p*	0.198	0.844
	LL (degree)	rho	0.082	0.006
		*p*	0.586	0.975
	PI (degree)	rho	0.069	−0.238
		*p*	0.650	0.232
	SS (degree)	rho	0.004	−0.316
		*p*	0.979	0.108
	PT (degree)	rho	0.105	−0.014
		*p*	0.487	0.944

**Table 4:** Spearman correlation coefficients. Coefficient values significant at *p* < 0.05 are marked in red.

Assessment of the Relationship between Variables, Spearman Correlations			
			C7 SVA HD (mm)
Spearman rho	OD-HA (mm)	rho	0.636
	abs OD-HA (mm)	rho	−0.029
	OD-HA (degree)	rho	0.602
	abs OD-HA (degree)	rho	0.023

## Data Availability

The data are available from the corresponding author if required.
